# Manufacture and Initial Characterisation of RAPID^TM^ Biodynamic Haematogel, an Autologous Platelet and Leukocyte-Rich Plasma Gel for Diabetic Foot Ulcers

**DOI:** 10.3390/gels10090572

**Published:** 2024-09-02

**Authors:** Aleksandra Olszewska, Jiajing Duan, Jana Javorovic, K. L. Andrew Chan, James Rickard, Simon Pitchford, Ben Forbes

**Affiliations:** 1 Institute of Pharmaceutical Science, King’s College London, Franklin-Wilkins Building, Stamford Street, London SE1 9NH, UK; aleksandra.olszewska@kcl.ac.uk (A.O.); ka_lung.chan@kcl.ac.uk (K.L.A.C.); simon.pitchford@kcl.ac.uk (S.P.); 2 Biotherapy Services Ltd., The Clarence Centre for Enterprise & Innovation, 6 St George’s Circus, London SE1 6FE, UK; james.rickard@biotherapyservices.com

**Keywords:** diabetic foot ulcers, platelet-rich plasma, diabetes, autologous therapeutics, point-of-care manufacture, wound healing

## Abstract

This observational study reports the process for the manufacture of RAPID^TM^ Biodynamic Haematogel and explores the properties of the platelet and leukocyte-rich plasma gels formed. Gels were manufactured from 60 mL of human blood using the protocol of Biotherapy Services. Platelet and leukocyte content, time-to-gel, gel weight and the temporal profile of liquid exudation from the gels were measured, along with the content of growth factors VEGF and PDGF in the releasate. The effect of the releasate on human keratinocyte (HaCat) cell proliferation was also determined. The platelet and leukocyte concentrations in donor blood were 1.60–8.10 × 10^8^ and 1.00 × 10^6^–2.00 × 10^7^ cells/mL, which were concentrated 2.67- and 1.12-fold, respectively, during processing. Structurally weak gels were formed which exuded a clear liquid releasate (77.4% *w*/*w* of gel weight over 60 min) that contained 278 pg/mL VEGF and 1319 pg/mL PDGF. The releasate produced concentration-dependent proliferation of HaCat cells: 5–15% releasate produced a 2.7–8.9-fold increase in growth over 48 h. In conclusion, we have described the point-of-care manufacturing protocol and characterised the gel properties of RAPID^TM^ Biodynamic Haematogel. This is an essential first step towards identifying, understanding and controlling critical processing parameters that impact on this medicinal product’s quality.

## 1. Introduction

### 1.1. Healthcare Burden of Diabetic Foot Ulcers

Diabetes is surging, and it is predicted that there will be between 1.22 and 1.39 billion diabetic patients globally by 2050 [1]. The lifetime risk of developing a diabetic foot ulcer (DFU) is 19–34% [2]. DFU is a life-altering or even life-threatening condition closely linked to other diabetes complications including neuropathy and vascular disease. The ulcer itself often originates from trauma to the foot, combined with insensitivity to pain, poor circulation and impaired healing processes in patients with diabetes, resulting in a hard-to-heal ulcer. Not only do DFUs significantly elevate mortality rate (2.48 times higher for diabetic patients with DFUs than without) [3], they also place an immense burden on global healthcare systems. The United Kingdom National Health Service spending on wound management was GBP 8.3 billion in 2017/2018, with GBP 5.6 billion directed towards unhealed wounds. Notably, 85% of unhealed wound costs were incurred in the community setting, of which only 6% was allocated to wound care products [4]. In the US, where an estimated 11.3% of the population lives with diabetes [5], DFUs were the second highest expense in wound care (after surgical wounds) reaching USD 9–13 billion in 2018 [6]. Pakistan, a country that in 2021 had the highest prevalence of diabetes worldwide (26.7%) [7], reports a pooled DFU prevalence of between 12–16% [8]. While no specific DFU-associated costs have been published, Pakistan experiences a significant economic burden of diabetes-associated treatments, reaching over USD 24 billion annually [9], and these numbers might underestimate the real costs as limited data are available.

### 1.2. Need for More Effective Treatments

The treatment and management of DFUs are challenging; the recurrence rate is 40% within the first year and as high as 65% within five years of the DFU healing [2]. Current management strategies revolve around multidisciplinary care in foot clinics, with podiatrists, nurses and vascular surgeons focusing on offloading, wound debridement, infection and pain management, and frequent dressing changes [10]. In severe cases, vascular reconstruction may be required or, in the absence of healing, amputation—an option associated with an almost 70% mortality rate for diabetic amputees within five years [2]. In an effort to develop more effective DFU treatments, the scientific and wider medical community is developing novel treatments, including advanced dressings and negative pressure therapy, as well as more personalised cellular therapies, e.g., stem cell, growth factor and platelet/fibrin-based therapies [11,12].

### 1.3. RAPID Gel as a Point-of-Care Therapeutic Product

Platelet-rich plasma (PRP) is a concentration of blood platelets which contains granules, the contents of which are associated with proliferative and potential wound healing benefits. Several growth factors found in PRP have been previously associated with tissue proliferation and wound healing. These include, but are not limited to, platelet-derived growth factor (PDGF) and vascular endothelial growth factor (VEGF) [13]. PRP can be generated from patient’s own blood and has potential as a regenerative therapy. It has been applied across many medical fields with mixed outcomes in terms of success, which has been attributed to poorly controlled preparation methods and lack of standardization. There have been relatively few large-cohort randomized controlled clinical trials (RCTs) [14,15]. Encouragingly, recent studies of PRP therapies for DFU have shown promising outcomes [16], despite utilising varying amounts of starting material, as well as different preparation methods, dosage forms, administration methods and frequencies.

RAPID Biodynamic Haematogel is an autologous leukocyte and platelet-rich plasma product that has been developed by Biotherapy Services. It is prepared using a point-of-care (POC) manufacturing process which enables doctors or healthcare professionals to manufacture the RAPID gel at a patient’s bedside. It has been developed through pilot studies and clinical trials in patients with DFU and has shown promising clinical outcomes [17]. As an autologous product, the safety concerns associated with blood and other biologically-derived products are avoided. POC manufacturing allows RAPID gel to be produced locally in clinical and home settings.

### 1.4. Challenges Associated with RAPID Gel

As a POC-manufactured autologous product, PRP offers benefits like ease of preparation and personalisation but also presents challenges. Variability in autologous products, such as growth factor content, is inevitable, and implementing stringent acceptance criteria for suitability of the blood used for product manufacture is impracticable. Rather, assurance of product quality can be achieved by control of the manufacturing process and definition of critical product attributes. Novel therapeutic PRP-based therapies such as RAPID gel are not currently recommended by the NHS, mostly due to a need for more comprehensive studies to establish benefit-to-cost margins [18,19]. Worldwide, these therapies are available for multiple conditions but are often ineligible for insurance coverage or only accessible as part of ongoing clinical trials [20]. Regulatory inconsistencies across countries further complicate issues like product standardization and best practices.

### 1.5. Quality Considerations for RAPID Gel

The quality of a unit dose for immediate-use POC therapy is difficult to ensure and constitutes a recognised challenge to current regulatory frameworks due to lack of a centralised manufacturing site and the usual GMP requirements and quality control processes that apply to pharmaceutical products. Instead, the product is made by different operators at multiple manufacturing sites. Moreover, the RAPID gel product is inherently variable due to inter-patient differences in blood characteristics, and assigning critical quality attributes to the gel is made difficult by the absence of definitive active agent(s) upon which to develop quality standards. To control quality and product consistency with all these challenges, the current approach is to control the manufacturing process itself and identify processing parameters that impact on critical product characteristics.

### 1.6. Aims and Objectives

This paper aims to define the manufacturing process for the RAPID gel and identify potential failure modes and critical processing parameters. Characterisation of the product will provide a first step towards developing analytical methods that can be used to probe the effects of processing parameters on putative crucial product attributes. This study will generate quantitative data for platelet and white blood cell content, thrombin-rich serum formation time, time-to-gel, and gel weight at the point of manufacture. It will also explore changes in the spectral fingerprint associated with gelation, the in vitro exudation of liquid releasate, along with releasate growth factor content and its effects on the proliferation of immortalized human keratinocytes. This constitutes the first report to provide complete end-to-end details of the manufacture method and associated product properties for RAPID Biodynamic Haematogel.

## 2. Results and Discussion

### 2.1. Reagent Preparation: L-PRP and Thrombin-Rich Serum

**Figure 1:**
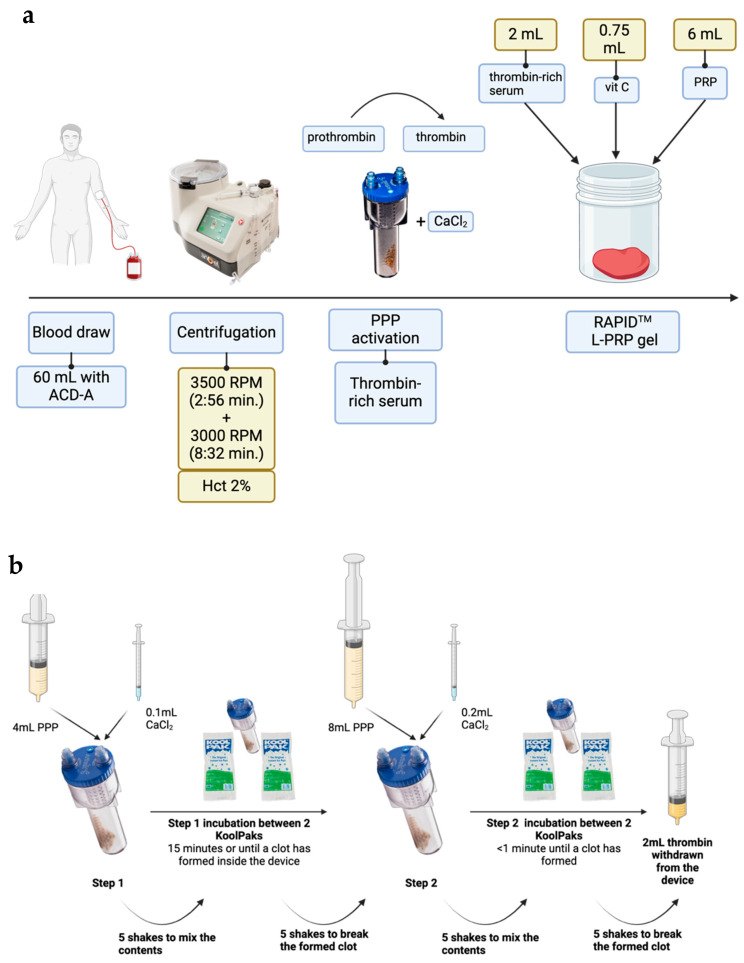
A summary of RAPID^TM^ Biodynamic Haematogel point-of-care (POC) manufacture (**a**) and a two-step process used to generate autologous human thrombin with the Thrombinator System (**b**). A pre-programmed two-step centrifugation in an Arthrex Angel machine is used to centrifuge whole blood, and the resultant platelet-poor plasma (PPP) is used to generate autologous thrombin-rich serum in a Thrombinator device. To manufacture a RAPID gel, L-PRP is combined with thrombin-rich serum and vitamin C. Created with BioRender.com. Abbreviations: Hct—haematocrit, PPP—platelet-poor plasma, L-PRP—leukocyte and platelet-rich plasma. Patent granted GB2579630.

The reagents to make a RAPID gel were produced from blood by concentrating platelets into L-PRP and using platelet-poor plasma (PPP) to make thrombin-rich serum. The concentration of platelets and white blood cells (WBC) in whole blood was 3.20 ± 1.84 × 10^8^ cells/mL and 6.61 ± 5.25 × 10^6^ cells/mL, respectively, (mean ± sd, *n* = 14). After two-step centrifugation in the Angel centrifuge (Figure 1) to produce L-PRP, platelets were concentrated 2.67 ± 0.86 times and leukocytes 1.12 ± 1.26 times, i.e., the platelet and leukocyte concentrations were 7.22 ± 3.90 × 10^8^ and 6.27 ± 9.38 × 10^6^ cells/mL, respectively, in L-PRP.

The platelet concentration in the L-PRP was higher than previously published data for the same process: 7.22 ± 3.90 × 10^8^ vs. 20.7 ± 5.26 × 10^7^ cells/mL [13]. However, the difference was not statistically significant (*p* = 0.22, unpaired *t*-test). In contrast, the WBC counts in this study were lower than those cited in the literature: 6.27 ± 9.38 × 10^6^ vs. 11.00 ± 4.5 × 10^6^ cells/mL (*p* = 0.64). It was also noted that the variability observed in the WBC counts in L-PRP was approximately twice as high as that observed previously [21]. These data illustrate that this first step in the manufacturing process is effective in selectively concentrating platelets, but it can also compound the variability in platelets and leukocytes in the starting material (whole blood). The separation is controlled by colorimetric sensors present in the machine, which recognise the ‘colour’ differences between centrifuged blood components rather than specific cells [22]. The manufacturer reports that there are three light beams present in the machine (470, 940 and 1300 nm), which pass through the processed blood differentially to detect different fractions (i.e., 940 nm will not pass through the red blood cell layer). The beams, however, do not recognise platelets and WBC separately when an L-PRP fraction is collected.

During the process of thrombin-rich serum production (Figure 1b), the time taken for the PPP within the Thrombinator device to form a clot, the ‘thrombin time’, was quantified as a process parameter that might be linked to product attributes. The thrombin time was 27 ± 7.6 min (*n* = 14, mean ± sd). The Thrombinator process is generally well controlled, although variation in the room temperature at which the thrombin-rich serum is generated is a factor that may have an impact. A greater source of variation may be the thrombin being produced as individuals have different levels of pro-thrombin, the starting component from which thrombin is generated during the process [23]. Further, thrombin is naturally degraded by anti-thrombin, which is also subject to inter-individual variation [24].

Overall, the preparative stages were completed within an hour. The length of time taken to generate the thrombin-rich serum and L-PRP to be ready to mix to produce the RAPID gel varied between 27 and 50 min, mainly depending on the time taken to generate the thrombin-rich serum.

### 2.2. RAPID—Gel Formation and Characterisation

**Figure 2:**
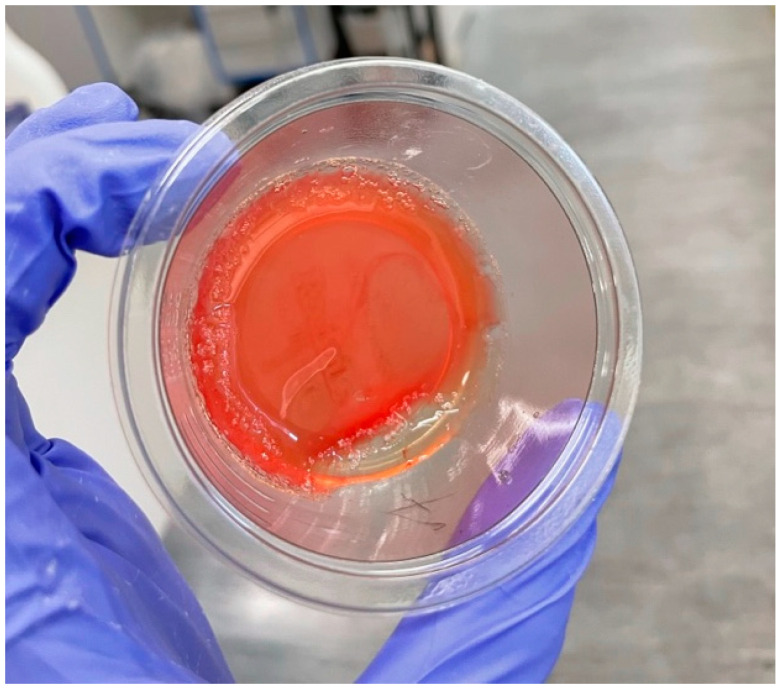
A representative image of a RAPID L-PRP gel.

**Figure 3:**
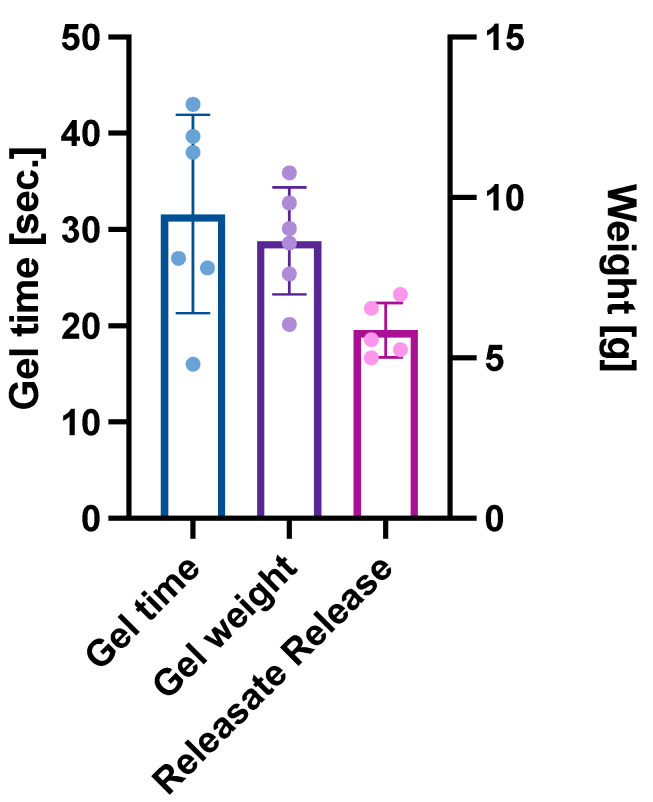
RAPID gel characteristics: (i) gel time—time taken for the formation of the gel after mixing of reagents, (ii) gel weight at the point of gel formation, and (iii) mass of liquid releasate exuded by the gels over 60 min in vitro (*n* = 6). Data are presented as mean ± sd, with individual data points shown as dots.

When manufacturing L-PRP gels using the Biotherapy Services protocol (Figure 1), it took 31 ± 10 s for gels to form and they weighed 8.65 ± 1.7 g (*n* = 6, mean ± sd). The gels were light red in appearance (Figure 2) and had a jelly-like form. The gels exuded 5.87 ± 0.85 g releasate in 60 min (Figure 3).

The time required for formation of the gel varied within a range (15–45 s), showing this step occurs quickly and is usually complete in less than a minute. Although the gel point was subjectively judged by the observer, the measurement accuracy is sufficient that the majority of the variation in the data is due to differences in time-to-gel, likely to be due to upstream differences in the manufacturing materials and process. The mechanism of RAPID gel formation is the development of a fibrin network upon platelet activation with thrombin [25,26]. The variability observed in gel time is likely due to a combination of factors associated with the blood donor, e.g., environmental factors such as gender, alcohol consumption, and the circadian rhythm, which affect autologous thrombin, the main activator of the gel formation.

The gels formed were relatively consistent in weight. As the process utilises volumetric instead of gravimetric measurements, the consistency of weight of each gel is dependent on the density of the thrombin and L-PRP suspensions and the accuracy of volumetric additions. Volumetric measurements are more practical in a point-of-care setting when different users carry out the manufacturing process at different sites. The use of single-use sterile syringes avoids the need for analytical balances but may sacrifice some accuracy.

**Figure 4:**
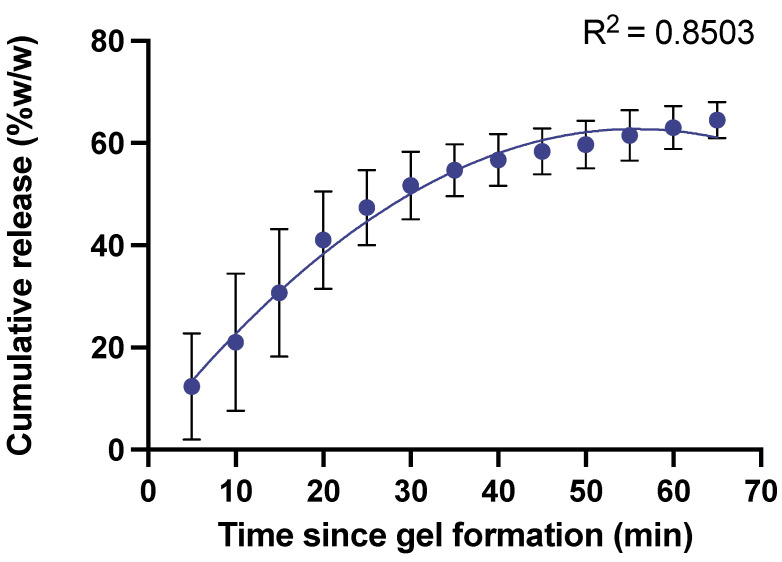
Cumulative loss of releasate from RAPID gels over time. A second order polynomial equation was fitted to model exudation of releasate by the gels. Data are presented as mean ± sd, *n* = 3. Data are presented as cumulative releasate mass exuded as a percentage of original gel weight. Time ‘zero’ is defined as the time the gel formation occurred following mixing of the reagents.

The mass of liquid releasate exuded in 60 min was reasonably reproducible (CV = 14.5%). The release profile shows the dynamic nature of RAPID gels, which from the point of formation, quickly began to exude fluid containing platelet lysate, with a temporal release profile that was consistent regardless of variability in initial gel weight (Figure 4). Through this process, the gel dried out during in vitro experiments in a way that may differ to a gel in situ within an occluded DFU.

The release profiles of the RAPID gels were fitted with a second order polynomial (quadratic) equation of the type commonly deployed to model response surface-based experiments [27,28]. The individual release profiles can be seen in Supplementary Materials. There was greater variability in the initial 15 min, but the gels consistently released 50% of their initial mass within 30 min of manufacture.

### 2.3. Gel Formation: FTIR Spectral Analysis

**Figure 5:**
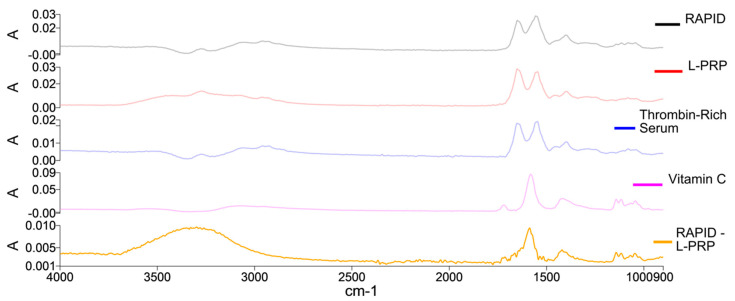
FTIR spectra of varying components of the RAPID gels starting with (top to bottom) the RAPID gels, Angel L-PRP, thrombin-rich serum, vitamin C and the spectrum of L-PRP subtracted from RAPID spectrum.

Fourier Transform Infrared Spectroscopy (FTIR) was performed on specially manufactured miniaturized gels to provide insights into the mechanism of formation of the RAPID gels. L-PRP samples were used as a control to compare their water-subtracted spectra to that of a RAPID gel (Figure 5).

The RAPID gel, L-PRP and thrombin-rich serum spectra show typical protein bands indicative of secondary protein structure, notably: amide I (peak at ~1650 cm^−1^), amide II (peak at ~1549 cm^−1^), as well as amide III with peak around 1235 cm^−1^; the remaining OH stretching is seen in the 3500–3000 cm^−1^ region, and CH stretching can be observed in the 2900 cm^−1^ region, which is also consistent with spectra observed for human blood cells, especially platelets [29]. The apparent difference when comparing the RAPID and L-PRP spectra reveals an absorbance pattern that resembles the spectrum of vitamin C (bottom spectrum on Figure 5, RAPID—L-PRP). Although the method can be used to detect the added component in the sample, it is not able to detect differences in gel formation mechanisms or gel properties.

### 2.4. Growth Factors in Releasate

In order to establish a connection between the properties of the gel and its therapeutic efficacy, the levels of two crucial growth factors involved in wound healing and tissue regeneration were measured in the fluid released from RAPID gels over a period of 60 min. The releasate contained 701 ± 395 pg/mL of VEGF and 3012 ± 1168 pg/mL of PDGF (mean ± sd, *n* = 3). Similar studies have been performed in the past on different PRP preparations, with similar findings pointing towards a burst release in the first hours, and growth factors showing a more ‘sustained’ release thereafter [30,31].

It is important to note that the gels may behave differently when in a moist wound environment. It has recently been shown that there are differences between ex vivo and in vitro growth factor release kinetic profiles, with ex vivo profiles (for human samples of wound fluid from tooth extraction wounds) being larger, which indicates varying behaviours depending on the environment [32].

### 2.5. HaCat Cell Proliferation over Time

The biological effect of RAPID gels was evaluated using a cell proliferation assay to measure the growth promoting effect of the releasate exuded from the gels over 60 min. The releasate was diluted in a supplement-free cell culture medium and added to human immortalized keratinocytes, HaCat cells. L-PRP was used as a control to compare its effects to those of the releasate.

**Figure 6:**
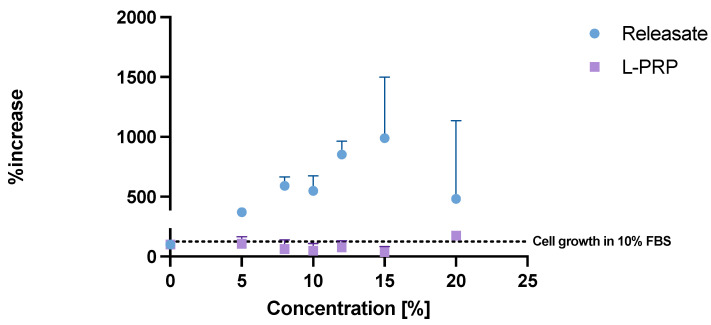
Immortalized human keratinocytes (HaCat) cell growth over a 48 h period in varying concentrations of L-PRP and RAPID releasate diluted in serum-free cell culture medium. Growth is measured as a relative fluorescence unit (RFU) detected upon the reduction of Resazurin by live cells during a 2 h incubation period. Data are presented as percentage increase in growth compared to the negative control, *n* = 3, mean + sd. A line has been added to show the growth of cells in DMEM medium with 10% foetal bovine serum. Abbreviations: NC—negative control (DMEM media w/o supplements); PC—positive control (DMEM w/10% foetal bovine serum); L-PRP—leukocyte and platelet-rich plasma.

The releasate produced concentration-dependent proliferation of HaCat cells: 5–15% releasate produced a 2.7–8.9-fold increase in growth over 48 h. However, this effect greatly varied between replicates at 15% (Figure 6). This may be explained by inherent variability in bioassay endpoints and the complex composition of the releasate, i.e., multiple growth factors with different dose–response relationships (as well as other bioactive compounds) being present in different concentrations and ratios in each releasate preparation.

Interestingly, in the in vitro assay, there appears to be a loss of the releasate proliferative effect at dilutions of 15–20% *v*/*v*. Further work is needed to follow up these initial observations. PPP, which is used in L-PRP and gel manufacture, is a mixture rich in proteins, among other molecules [33], and in contrast to our findings, has been previously shown to have an effect on cell proliferation [34]. The in vitro effects in Figure 6 require confirmation in cells besides keratinocytes, e.g., fibroblasts, which contribute importantly to wound healing and more complex primary cell culture systems, and future studies might include wound-healing assays under normal and high (i.e., diabetes-simulating) glycemic conditions. Further, the Resazurin assay is a measure of cell metabolism, which is positively correlated with cell number, but requires verification that the effects are associated with increased cell number.

### 2.6. Future Work

Collectively, these results are a first step towards linking key aspects of the RAPID gel manufacturing process to the product formed and its efficacy. There is inevitable variability associated with the use of autologous blood as the starting material, and inter-individual differences in blood composition are hypothesised to influence therapeutic efficacy and may contribute to the variation observed in growth factor release and the HaCat cell proliferation assay. It would be interesting to study how the therapeutic efficacy of the gel is influenced by the kinetics and duration of growth factor availability produced in the wound site. Tighter criteria could be introduced regarding the starting material. The medical history of the patient needs to be carefully considered as many commonly used medications are known to impact platelet activity and might have an impact on the quality and efficacy of a RAPID gel. Clarification of how platelet and white cell counts in blood affect L-PRP gel quality and efficacy would enable specifications to be set for prerequisite cell concentrations when embarking on RAPID gel therapy. The implications of using of healthy donor rather than autologous patient blood on product efficacy is another interesting consideration for future studies.

As a POC product, assurance of product quality requires a robust and reproducible manufacturing protocol in which the critical processing parameters are identified and controlled. For example, the time to generate the thrombin-rich serum impacts the overall manufacturing time and needs to be further investigated. Other aspects for attention to ensure control of the manufacturing process include tighter environmental control (temperature control) during the manufacturing, and perhaps introduction of pass/fail criteria together with a full failure mode and effects analysis. The next steps are to determine the critical processing parameters and critical quality attributes of the RAPID gels to control the manufacturing process and ensure the quality and efficacy of the final product.

### 2.7. A Note on Regulatory Framework for POC Products

An important consideration for blood-based products is their regulatory position. Adding to the lack of standarization and multiple preparation methodologies are the multiple regulatory stances. For example, in the US, PRP is not fully regulated by the FDA but can be used off-label in healthcare clinics. While PRP lacks full FDA approval, it is considered an emerging technology. Current regulations allow for POC manufacture of PRP in clinics, excluding use during surgeries, leading to high costs and limited insurance coverage. Liability concerns further restrict its provision by healthcare professionals [35]. In the European Union, PRP is governed by the blood directive and the class II medical device directive, yet regulations vary by country, leading to inconsistent regulatory landscapes. For instance, PRP is classified differently in Spain, Italy, and the Netherlands, with varying degrees of regulatory oversight [36,37]. The UK’s MHRA currently has strict rules on PRP use and classifies them as medicinal products if medical use is intended, meaning that PRP and its derivatives need to comply with the Human Medicines Regulations. At present, PRP products are not recommended for indications such as DFU due to limited data and relatively high costs [18]. However, the MHRA recognises that autologous products might not fit well within the current regulatory structure and is actively developing a novel regulatory framework for POC manufacturing, which would include PRP, aimed at standardizing safety and quality controls while fostering innovation in the medical product market [38].

## 3. Conclusions

In conclusion, the RAPID gel manufacturing protocol is reported with quantitative characterisation of the resultant leukocyte and platelet-rich plasma gels. Releasate from the L-PRP gels contains the products of platelet activation and showed a significant increase in keratinocyte proliferation rates compared to L-PRP alone. As a POC autologous blood product, variability in the starting material is inevitable, and assurance of product quality requires control of the manufacturing process by identifying critical starting material attributes and processing parameters that impact critical quality attributes to produce a clinically suitable product for each patient.

## 4. Materials and Methods

### 4.1. Blood Collection and Cell Counts

Informed consent was obtained from each blood donor. All procedures complied with King’s College London local ethical approval (ref: HR/DP-23/24-28260) and the regulations outlined by Human Tissue Act 2004. For each donation, 60 mL of peripheral whole blood was drawn from medial cubical vein of healthy volunteers into anticoagulant citrate dextrose solution A—ACD-A—prefilled 9 mL Vacuette^®^ tubes (Greiner Bio-One Ltd., Stonehouse, UK).

To measure platelet and white blood cell (WBC) counts, 2 μL of pre-processed whole blood was removed from the blood collection tube, mixed with 198 μL of Stromatol (Mascia Brunelli S.p.A, Milan, Italy) and counted using a haemocytometer to determine platelet and WBC concentrations. A reference range of 1.5–4.0 × 10^8^ cells/mL platelets and 4.5–11 × 10^3^ cells/mL for WBC was regarded as the acceptable limit [39]. One sample was rejected due to a donor having a lower platelet count in whole blood. After centrifugation, 2 μL of L-PRP generated from the Angel machine was removed, mixed with Stromatol and counted in the same manner as above to determine cell counts in the L-PRP.

### 4.2. RAPID^TM^ Gel Manufacture

Standard Biotherapy Services ‘Chronic’ Protocol (2% haematocrit, ref: BTS-BMR-003/Patent GB2579630) was followed for RAPID gel manufacture (Figure 1). Immediately following collection, blood was centrifuged for 12:38 min in total (3500 RPM for 2:56 min, followed by 3000 RPM for 8:32 min) in an Arthrex Angel^TM^ centrifuge (Arthrex Angel^TM^ System, Arthrex Gmbh, Munich, Germany, cat no: ABS-10064). Centrifugation produced an L-PRP fraction of approximately 2.5 ± 0.5 mL and a platelet-poor plasma (PPP) fraction of approximately 25.9 ± 4.0 mL.

The L-PRP fraction was diluted to 6 mL with PPP and platelet, and WBC concentrations were measured as described above. The PPP was used to form the thrombin-rich serum using a Thrombinator System (Arthrex Gmbh, Munich, Germany) and the two-step protocol of the manufacturer (Figure 1b). The reaction was performed at room temperature, and the thrombin time (time from the start of step 1 of the reaction to the final clot and subsequent withdrawal of the serum) was recorded for each donor.

To form the RAPID gel, 6 mL of L-PRP was mixed by swirling the cup 3 to 5 times with 2 mL of thrombin-rich serum and 0.75 mL of ascorbic acid (500 mg/5 mL, Sanorell Pharma GmbH, Bühl/Baden, Germany). The product was weighed at the point of manufacture and the time to gel measured using a stopwatch.

Miniaturized gels for FTIR analysis were manufactured by combining the same reagents in the same ratio but in lower quantities (494 μL L-PRP, 62 μL vitamin C, 162 μL thrombin-rich serum) in order to fit the stage of the FTIR apparatus.

### 4.3. RAPID L-PRP Gel Characterisation

#### 4.3.1. Exudation of Releasate and Growth Factor Measurement

To measure the rate at which releasate (fluid exuded from RAPID gels) was produced, gels from three donors were analysed every 5 min for 60 min. The releasate was collected by carefully aspirating all the released liquid using a Gilson pipette at each given timepoint and quantifying it gravimetrically. The release profile was plotted cumulatively as percentage of the mass of the original gel as a product of time (Figure 4).

The releasate collected over 60 min was pooled, frozen using dry ice and stored at −20 °C until assayed for growth factor concentration. Human vascular endothelial growth factor (VEGF) and platelet-derived growth factor BB (PDGF-BB) Sandwich Enzyme-linked immunosorbent Assay (ELISA) kits were used (Quantikine, Elisa Kit, R&D Systems, Abingdon, UK) to quantify growth factors. The manufacturer’s protocol was followed to quantify the growth factor content in L-PRP and the gel releasate.

#### 4.3.2. Fourier Transform Infrared Spectroscopy (FTIR)

For the FTIR, miniaturized gels were prepared as described above following the original protocol reagent ratios. L-PRP and RAPID gel samples were prepared on a 6-well plate and carefully transferred using forceps onto the ATR Diamond Accessory (Specac Ltd., Orpington, UK). Spectral data were collected using an FTIR Spectrum One spectrometer (PerkinElmer, Beaconsfield, UK) over a 4000 to 900 cm^−1^ spectral range. This is a mid-IR range that contains the sharpest absorbance peaks, providing the best opportunity to identify differences between gels if they are detectable. Due to the fast-changing nature of the samples, for the RAPID gel scans, 16 accumulations were chosen with an 8 cm^−1^ spectral resolution. Preliminary experiments conducted on similar samples showed that the peak sharpness does not improve if higher spectral resolution is selected, but it does take longer for the spectra to be obtained. A 0.9% sodium chloride in water solution was analysed as a background (using 32 accumulations and 8 cm^−1^ spectral resolution) to enable water peaks to be removed in spectral post processing. This solution was chosen as the gel samples are mostly composed of water, the absorbance of which needs to be subtracted as it dominates the FTIR spectrum of the gel and masks the spectral features of interest. NaCl is infrared transparent, so adding NaCl does not produce interfering bands and provides an ionic strength of solution similar to that of the gel.

### 4.4. Keratinocyte Proliferation Assay

Human immortalized keratinocytes, HaCat cells, were cultured in T75 flasks (Nunc, ThermoFisher Scientific, Loughborough, UK) in high glucose Dulbecco’s Modified Eagle’s Medium (DMEM) media (Gibco, ThermoFisher Scientific, Loughborough, UK) supplemented with 10% *v*/*v* foetal bovine serum (FBS) (Merck Life Science Ltd., Dorset, UK) and 1% penicillin/streptomycin mix (10,000 U/mL, ThermoFisher Scientific). The flasks were maintained in a humidified incubator (Culture Safe Precision CO_2_, LEEC, Nottingham, UK) at 37 °C and 5% CO_2_. The cells were routinely subcultured when ~60–70% confluency was reached.

For the proliferation assay, HaCat cells were seeded at a 2000 cells/well concentration in two 96-well plates (Greiner Bio-One) for L-PRP and releasate, respectively, in 100 μL of supplement-free DMEM media and incubated for 24 h to allow the cells to attach. The gel releasate was collected as described in Section 4.3.1 and diluted with DMEM phenol red-free (Gibco, ThermoFisher Scientific) to provide test samples of 5–20% *v*/*v* gel releasate. Equivalent dilutions of L-PRP were prepared to enable comparison with a non-activated platelet preparation. Cells were exposed to the releasate and L-PRP samples for 24 h, and then quantified using the Resazurin assay.

Resazurin (R&D Systems, Abingdon, UK) was added to each well in a 10% concentration (11 μL to 100 μL media) and the plates were returned to the incubator for 2 h to allow the cells to reduce Resazurin. The fluorescence reading was carried out with a plate reader (Spark^®^, Tecan, Scientific Laboratory Supplies Ltd., Nottingham, UK) by setting the excitation wavelength to 535 nm and the emission wavelength to 595 nm.

### 4.5. Data Analysis

The data were analysed using Microsoft Excel (Version 16.87) and GraphPad Prism 10. All data are presented as mean ± sd unless stated otherwise. All variables and data sets were subjected to a normality test (Shapiro–Wilk) prior to further statistical analysis.

## Data Availability

The datasets presented in this article are not readily available because this research includes human blood samples, thus data is protected by GDPR. Requests to access parts of the datasets should be directed to Corresponding Authors.

## Supplementary Materials

The following supporting information can be downloaded at: https://www.mdpi.com/article/10.3390/gels10090572/s1, Figure S1 Cumulative loss of releasate from three RAPID gels over time. A second order polynomial equation was fitted to model exudation of releasate by the gels. Data are presented as individual repeats, n = 3. Data are presented as cumulative releasate mass exuded as a percentage of original gel weight. Time ‘zero’ is defined as the time the gel formation occurred following mixing of the reagents.
