# Supplementary material for: Manufacture and Initial Characterisation of RAPIDTM Biodynamic Haematogel, an Autologous Platelet and Leukocyte-Rich Plasma Gel for Diabetic Foot Ulcers
Source: Gels. 2024 Sep 2;10(9):572. doi: 10.3390/gels10090572 (PMC11431608; doi:10.3390/gels10090572)
Supplement: Supplementary file 1 [file gels-10-00572-s001.zip › gels-3093907-supplementary.pdf]

# Supplementary Materials

# Manufacture and Initial Characterisation of RAPID™ Biodynamic Haematogel, an Autologous Platelet and Leukocyte-Rich Plasma Gel for Diabetic Foot Ulcers

**Aleksandra Olszewska [1], JiaJing Duan [1], Jana Javorovic [1], K. L. Andrew Chan [1], James Rickard [2], Simon Pitchford [1] and Ben Forbes [1,*]**

[1] Institute of Pharmaceutical Science, King's College London, Franklin-Wilkins Building, Stamford Street, London SE1 9NH, UK; aleksandra.olszewska@kcl.ac.uk (A.O.); ka_lung.chan@kcl.ac.uk (K.L.A.C.); simon.pitchford@kcl.ac.uk (S.P.)
[2] Biotherapy Services Ltd., The Clarence Centre for Enterprise & Innovation, 6 St George's Circus, London SE1 6FE, UK; james.rickard@biotherapyservices.com
* Correspondence: ben.forbes@kcl.ac.uk

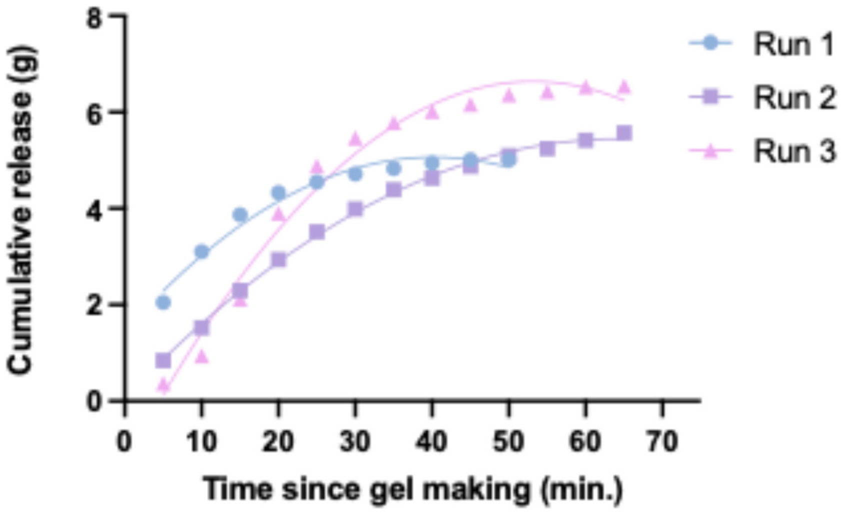


**Figure S1**. Cumulative loss of releasate from three RAPID gels over time. A second order polynomial equation was fitted to model exudation of releasate by the gels. Data are presented as individual repeats, n = 3. Data are presented as cumulative releasate mass exuded as a percentage of original gel weight. Time 'zero' is defined as the time the gel formation occurred following mixing of the reagents.
